# Ustekinumab in Paediatric Patients with Moderately to Severely Active Crohn’s Disease: Pharmacokinetics, Safety, and Efficacy Results from UniStar, a Phase 1 Study

**DOI:** 10.1093/ecco-jcc/jjab089

**Published:** 2021-05-26

**Authors:** Joel R Rosh, Dan Turner, Anne Griffiths, Stanley A Cohen, Douglas Jacobstein, Omoniyi J Adedokun, Lakshmi Padgett, Natalie A Terry, Christopher O’Brien, Jeffrey S Hyams

**Affiliations:** 1 Pediatric Gastroenterology, Goryeb Children’s Hospital, Morristown, NJ,USA; 2 Pediatric Gastroenterology, Shaare Zedek Medical Center, Hebrew University of Jerusalem, Jerusalem, Israel; 3 Pediatric Gastroenterology, Hospital for Sick Children, University of Toronto, Toronto, ON, Canada; 4 Pediatric Gastroenterology, Children’s Center of Digestive Health Care, Atlanta, GA, USA; 5 Provention Bio, Inc., Redbank, NJ, USA; 6 Janssen Research and Development LLC, Spring House, PA, USA; 7 Pediatric Gastroenterology, Connecticut Children’s Medical Center, Hartford, CT, USA

**Keywords:** Crohn’s disease, ustekinumab, paediatric

## Abstract

**Background and Aims:**

The objective was to evaluate the pharmacokinetics, safety/tolerability, and efficacy of ustekinumab in children with moderately to severely active Crohn’s disease.

**Methods:**

In this Phase 1, multicentre, 16-week, double-blind, induction dose-ranging study [NCT02968108], patients aged 2‐<18 years [body weight ≥10 kg] were randomised [1:1] to one of two weight range-based intravenous induction doses: 130 mg vs 390 mg in patients ≥40kg and 3 mg/kg vs 9 mg/kg in patients <40kg. At Week 8, all patients received a single subcutaneous ustekinumab maintenance dose of 90 mg in patients ≥40kg or 2 mg/kg in patients <40kg.

**Results:**

A total of 44 patients were randomised and treated with ustekinumab [*n* = 23 lower dose; *n* = 21 higher dose]; median [interquartile range] age was 13.0 [12–16] years. Pharmacokinetics were similar to those in adults with Crohn’s disease. However, serum ustekinumab concentrations were lower among those with body weight <40 kg compared with patients ≥40 kg and the reference Phase 3 adult population. Through Week 16, 73% of patients reported ≥1 adverse event [82.6% lower vs 62% higher dose]; two discontinued due to adverse events [one in each group]. Serious adverse events occurred in 16% [26% lower, 5% higher dose], with Crohn’s disease exacerbation being the most frequent. At Week 16, 22%/29% [lower/higher dose] achieved clinical remission [Paediatric Crohn’s Disease Activity Index ≤10].

**Conclusions:**

The pharmacokinetics/safety profiles were generally consistent with those observed in adults with Crohn’s disease. These results suggest a different dosing regimen may be required for patients <40 kg from that employed in this study; additional pharmacokinetic analyses may be needed in this population.

## 1. Introduction

Childhood-onset Crohn’s disease [CD] is most commonly ileocolonic; often evolves into complicated disease phenotypes; has high prevalence of extraintestinal manifestations; and, despite the use of immune-modifying therapies, often requires surgical resection.^1^ In addition, children are more frequently exposed to steroids, which can lead to dependence.^2^

Currently, there are only two approved biologic therapies [both tumour necrosis factor [TNF] antagonists] for the treatment of paediatric CD in North America and the European Union. As in adults, a sizeable portion of children respond initially and then lose response or become intolerant to current treatments, including TNF-antagonist therapies.^3^ Thus, there is a considerable unmet medical need for novel therapeutic options that are safe, effective, and convenient for children with CD.

Ustekinumab is a first-in-class fully human immunoglobulin G1κ monoclonal antibody that binds with specificity to the p40 protein subunit of interleukins [IL]-12 and IL-23. These cytokines are involved in inflammatory and immune responses such as natural killer cell activation and CD4+ T cell differentiation and activation.^4^

Ustekinumab is indicated for the treatment of moderately to severely active CD, ulcerative colitis, and active psoriatic arthritis in adults, as well as moderate to severe plaque psoriasis in adults and children.^4^ The efficacy and safety of ustekinumab in adults with CD have been previously established in the UNITI-1 and UNITI-2 induction studies and in the IM-UNITI maintenance study.^5^ Long-term ustekinumab maintenance therapy has been evaluated in the IM-UNITI long-term extension studies through 2 and 3 years.^6,7^

In this Phase 1 study, we evaluated the pharmacokinetics [PK], safety/tolerability, and efficacy of ustekinumab in paediatric patients with moderately to severely active CD. Results through Week 16 are presented here; the long-term extension [LTE] is ongoing.

## 2. Materials and Methods

### 2.1. Study design

**Figure 1.**
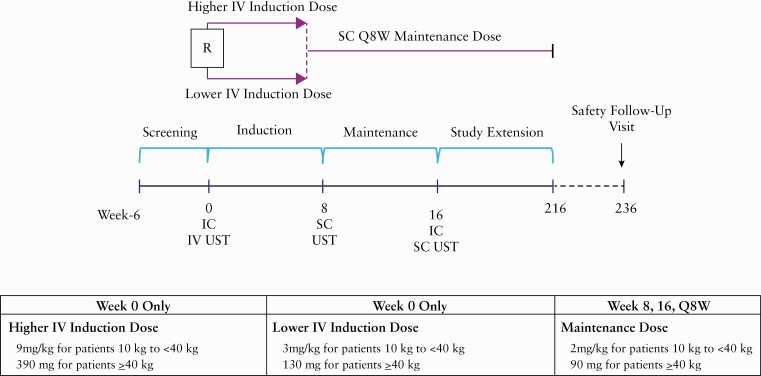
UniStar study design. IC, ileocolonoscopy; IV, intravenous; Q8W, every 8 weeks; R, randomized; SC, subcutaneous; UST, ustekinumab.

UniStar is a randomised, double-blind, dose-ranging study of intravenous [IV] ustekinumab induction followed by subcutaneous [SC] ustekinumab maintenance in paediatric patients with moderately to severely active CD **[**Figure 1**].** Within the 2 weeks preceding Week 0, patients underwent an ileocolonoscopy, and at Week 0, patients were randomised 1:1 into one of two treatment groups stratified by weight [<40 kg or ≥40 kg] and previous TNF-antagonist exposure status.

### 2.2. Dosing

The dosing regimens evaluated in this study were modelled to deliver ustekinumab exposure comparable to that observed in the corresponding reference adult CD population (ie, UNITI-1 [NCT01369329], UNITI-2 [NCT01369342], and IM-UNITI [NCT01369355] studies).^5,8^ Patients in each treatment group received a single IV induction dose of ustekinumab at Week 0 as follows:

- lower IV induction dose [herein referred to as ‘lower dose’] of 3 mg/kg for patients 10 kg to <40 kg body weight or 130 mg for patients ≥40 kg body weight;
- higher IV induction dose [herein referred to as ‘higher dose’] of 9 mg/kg for patients 10 kg to <40 kg body weight or 390 mg for patients ≥40 kg body weight.

All patients then received an SC dose of ustekinumab at Week 8: 2 mg/kg for patients 10 kg to <40 kg or 90 mg for patients ≥40 kg. At Week 16, patients underwent a second ileocolonoscopy and were eligible to enter the LTE, where they received the following maintenance doses of SC ustekinumab every 8 weeks: 2 mg/kg for patients 10 kg to <40 kg or 90 mg for patients ≥40 kg.

### 2.3. Patient population

Eligible patients were 2 to <18 years old in the USA, 6 to <18 years old elsewhere, of either sex, with a body weight of ≥10 kg and a diagnosis of moderately to severely active CD or fistulising CD for ≥3 months, with active colitis, ileitis, or ileocolitis confirmed at any time in the past by radiography, histology, and/or endoscopy. Inclusion criteria for patients in this study included a baseline Paediatric CD Activity Index [PCDAI] score of >30, in addition to at least one of the following: an abnormal C-reactive protein [CRP] value [>0.3 mg/dL] at screening [72.7%]; faecal calprotectin of >250 µg/g at screening [88.1 %]; or an ileocolonoscopy with evidence of active CD [defined as ulcerations in the ileum and/or colon] during screening/baseline visit [84.1%]. Seven patients who did not have a simple endoscopic score for CD [SES-CD] ≥3 or ulcerations at baseline were included in the study. The protocol for this study was modelled on previous adult studies of ustekinumab in CD.^5–8^ All patients received previous or current medication for CD including oral corticosteroids and/or the immunomodulators azathioprine [AZA], 6-mercaptopurine [6-MP], or methotrexate [MTX]. Patients were required to be stable for at least 2 weeks on steroids before study start and stable for at least 4 weeks on immunomodulators before study start. Those who failed or were intolerant to anti-TNF therapy were eligible for participation. However, patients with exposure to anti-TNF biologic agents within 8 weeks of baseline and vedolizumab within 16 weeks of baseline were not eligible for entry into the study.

Steroid tapering was left to the discretion of the investigator and could begin at Week 3 to allow more flexibility in the protocol. The protocol did specify that enrolled patients should not initiate or increase the dose of any of the following concomitant CD-specific medical therapies: oral 5-aminosalicylates [ASA], oral corticosteroids, immunomodulators [i.e. AZA, 6-MP, or MTX], and/or total parental or enteral nutrition up to Week 16. Patients who did initiate or increase the dose of these specific medical therapies up to Week 16 were considered treatment failures.

### 2.4. Outcomes

Ustekinumab serum concentrations and anti-drug antibodies [ADAs] were assessed. Serum ustekinumab concentrations and ADAs were evaluated using a validated drug-tolerant electrochemiluminescent immunoassay method on the Meso Scale Discovery [MSD®] platform [Gaithersburg, MD]. ADAs were detected in up to 100 μg/mL of ustekinumab without interference. Safety was assessed by summarising the frequency and type of AEs.

The PCDAI is a validated multi-item measure of disease activity.^9^ Clinical response [reduction from baseline in PCDAI score of ≥15 points] and remission [defined as a PCDAI score ≤10 points] at Week 16 was assessed. Daily diaries one week preceding scheduled visits were collected for abdominal pain, stool pattern, and general well-being [instead of 1-week history recall]. Diaries from the date of bowel preparation to the date of the procedure and the day after the procedure were excluded in the PCDAI calculation. The worst item [defined by the highest sub-score] across 7 days of non-excluded diaries for each sub-component were used for that visit. The patient had to complete four or more non-excluded evening diaries to calculate the history components of the PCDAI score.

Ileocolonoscopies were conducted at baseline and Week 16. Endoscopic response [reduction in SES-CD ≥50%] and remission [SES-CD ≤2] were assessed. To evaluate the SES-CD endpoints, patients with evaluable endoscopy data were considered eligible for the SES-CD analyses if they had a baseline SES-CD ≥3, excluding the contribution of the narrowing component score. These exclusion criteria were designed to ensure that there was active disease reached by endoscopy, not just a high score due to stricture.

A single reader at a central facility evaluated and scored all video endoscopies. The reader was blinded to treatment group throughout the duration of the study. Patients with at least one segment evaluated were considered to have evaluable endoscopy data. The reader recorded whether the endoscopy was evaluable or not evaluable, due to poor bowel preparation or other technical reasons. For each evaluable endoscopy, the reader scored each ileocolonic segment using the SES-CD and recorded the presence or absence of mucosal ulceration for each segment.

### 2.5. Statistical analysis

A sample size of 40 patients was chosen empirically for PK assessments based on experience from previous PK studies of other biologics in paediatric patients, including the study of golimumab in paediatric patients with ulcerative colitis.^10^ Given the moderate variability associated with ustekinumab PK parameters in adult patients with CD and assuming comparable PK variability between adult and paediatric patients, a sample size of 40 was considered sufficient to provide adequate PK data in paediatric patients with CD. UniStar was considered to have met its primary objective if the PK of ustekinumab in paediatric patients with CD was demonstrated to be consistent with that previously observed in the reference adult CD population.

No formal hypothesis testing was conducted. Descriptive statistics [e.g., mean, median, standard deviation, interquartile range, minimum, and maximum] were used to summarise continuous variables. Counts and percentages were used to summarise categorical variables.

Patients who had any of the following events were considered to be treatment failures from the time of event onward: [1] a CD-related surgery thought to be a result of lack of efficacy of study agent [with the exception of minor procedures such as drainage of a superficial abscess or seton placement]; [2] discontinued study agent due to an adverse event [AE] of worsening CD or due to lack of efficacy; or [3] specified changes in concomitant CD medications.

Treatment failure rules were applied for all efficacy endpoints unless otherwise specified. In such a case, baseline values [at Week 0] were assigned from the point of treatment failure onward through Week 16, regardless of the observed data, for continuous endpoints and patients were considered as not achieving the respective endpoints for dichotomous endpoints. Treatment failure rules overrode other data handling rules.

For all analyses, patients with insufficient data for binary endpoints were considered to not have achieved their respective endpoint; for patients with insufficient data for continuous endpoints, the last available value was carried forward. These data handling rules were applied for all efficacy endpoints.

The total PCDAI score was calculated when three or more of the five components were available in full [i.e., all sub-components were complete] from the visit where the PCDAI was measured. If less than three components were completely available, then the PCDAI was not calculated based on the information collected at the visit. Conditional on three or more of the five component scores being available from the visit where the PCDAI was to be measured, in general, when the value of a sub-component within each of the five components was missing, the closest previous sub-component value was used. If all the sub-component scores of a component were missing, the closest previous component value was used.

## 3. Results

A total of 44 patients were randomised and treated with ustekinumab: 23 in the lower induction dose group [ustekinumab 3 mg/kg IV or 130 mg IV] and 21 in the higher induction dose group [ustekinumab 9 mg/kg IV or 390 mg IV; Supplementary Figure 1, available as Supplementary data at *ECCO-JCC* online]. The 44 patients who were randomised and treated were from 15 sites as follows: 65.9% [29 patients] were from Belgium, France, Germany, and Poland; 34.1% [15 patients] were from the USA and Canada. Among these, 59.1% were female, 81.8% were White, and median age and weight were 13.0 years (interquartile range [IQR] 12–16 and 42.9 kg, [IQR 35–52], respectively **[**Table 1**]**.

**Table 1.** Summary of baseline characteristics.^a^

	Lower dose UST	Higher dose UST	Combined
	3 mg/kg IV or 130 mg IV	9 mg/kg IV or 390 mg IV	
Patients treated	23	21	44
Age [years]	13 [11–16]	13 [12–16]	13 [12–16]
Sex, female	17 [74]	9 [43]	26 [59]
Weight [kg]	43 [35–54]	43 [31–51]	43 [35–52]
<40 kg, *n* [%]	10 [44]	8 [38]	18 [41]
≥40 kg, *n* [%]	13 [57]	13 [62]	26 [59]
CD duration [years]	4 [2–5]	4 [2–8]	4 [2–6]
PCDAI score	43 [40–53]	43 [34–50]	43 [38–50]
SES-CD	18 [8, 23]	15 [13, 19]	15 [11, 23]
Previous/current treatments			
Previous biologic exposure	21 [91]	19 [91]	40 [91]
Infliximab	17 [74]	16 [76]	33 [75]
Adalimumab	13 [57]	12 [57]	25 [57]
Vedolizumab	1 [4]	1 [5]	2 [5]
Concomitant medications for CD	19 [83]	13 [62]	32 [73]
Immunomodulators^b^	7 [30]	10 [48]	17 [39]
Oral corticosteroids	7 [30]	7 [33]	14 [32]
Oral aminosalicylates	4 [17]	5 [24]	9 [21]
Antibiotics	2 [9]	0	2 [5]
Location of disease, *n*	*n* = 23	*n* = 20	*n* = 43
Ileum only	4 [17]	1 [5]	5 [12]
Colon only	8 [35]	6 [30]	14 [33]
Ileum and colon	11 [48]	13 [65]	24 [56]
Severity of disease	*n* = 22	*n* = 20	*n* = 42
Remission/mild disease [PCDAI ≤30]	1 [5]	4 [20]	5 [12]
Moderate disease [PCDAI >30-≤40]	5 [23]	4 [20]	9 [21]
Severe disease [PCDAI >40]	16 [73]	12 [60]	28 [67]
Biomarkers of inflammation^c^	*n* = 23	*n* = 21	*n* = 44
CRP [mg/L]	16 [5–34]	8 [2–22]	13 [2–30]
Faecal calprotectin [mg/g]	2907 [1596–4772]	1814 [710–3007]	2210 [807–3900]
Faecal lactoferrin [µg/mL]	232 [49–376]	123 [65–267]	164 [63–345]

CD, Crohn’s disease; CRP, C-reactive protein; IV, intravenous; PCDAI, Pediatric Crohn’s Disease Activity Index; SES-CD, simple endoscopic score for Crohn’s disease; UST, ustekinumab.

^a^Dichotomous variables expressed as *n* [%] and continuous variables expressed as median [interquartile range].

^b^Azathioprine [AZA], 6-mercaptopurine [6-MP], or methotrexate [MTX].

^c^Not all patients had each test.

Baseline disease characteristics were representative of patients with moderately to severely active CD and were generally well balanced across the two treatment groups in age, weight [41% <40 kg; 59% ≥40 kg], median duration of disease at baseline [3.6 years; IQR 2–6], and median PCDAI score [43; IQR 38–50]. However, baseline median CRP, faecal lactoferrin, and calprotectin concentrations were numerically greater in the lower than in the higher dose group, and there were more female patients in the lower dose group.

Forty patients [91%] had previous exposure to biologics at baseline, including 33 [75%] who had received infliximab and 25 [57%] who had received adalimumab. Ten patients [22.7%] had an inadequate initial response to TNF antagonists, 26 [59.1%] had a response followed by a loss of response, and 13 [29.5%] had intolerance to one or more TNF antagonists. A total of 24 patients [54.5%] had failed one TNF antagonist agent, and 15 [34.1%] had failed two TNF antagonist agents. Additionally, 73% of patients were receiving one or more other medication prescribed for CD therapy at baseline. The proportions of patients receiving each type of CD medication were generally similar between treatment groups (39% immunomodulators, 21% oral aminosalicylates, and 32% oral corticosteroids [including budesonide]).

Four of the 44 treated patients [9.1%] discontinued study agent through Week 16; twp of these were due to AEs [both worsening of CD]. The remaining two discontinued due to lack of efficacy and investigator’s decision.

### 3.1. Pharmacokinetics

**Figure 2.**
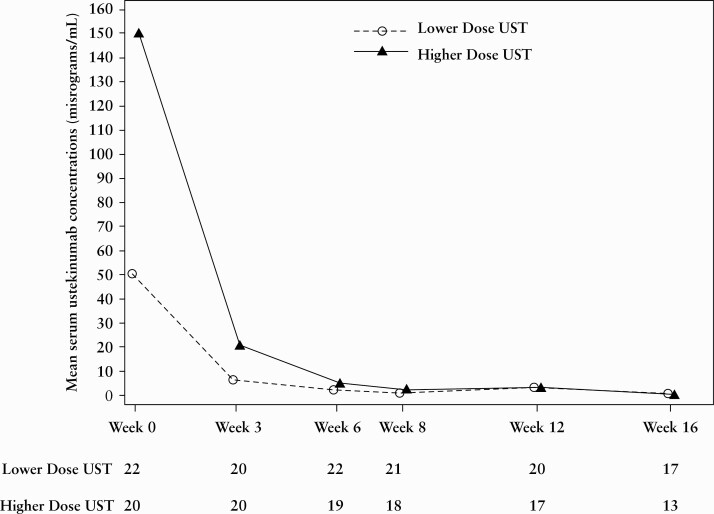
Line plot of mean serum UST concentrations [μg/mL] through Week 16. BW, body weight; IV, intravenous; UST, ustekinumab; lower dose, 3 mg/kg IV for patients <40 kg BW or 130 mg IV for patients ≥40 kg BW; higher dose, 9 mg/kg IV for patients <40 kg BW or 390 mg IV for patients ≥40 kg BW.

At Weeks 0 [1 h after infusion], 3, 6, and 8, mean serum ustekinumab concentrations [SUCs] in the lower [51.3, 7.7, 3.0, and 1.6 μg/mL, respectively] and higher dose groups [149.0, 23.7, 9.1, and 4.8 μg/mL, respectively] were generally dose proportional **[**Figure 2**]**.

**Figure 3.**
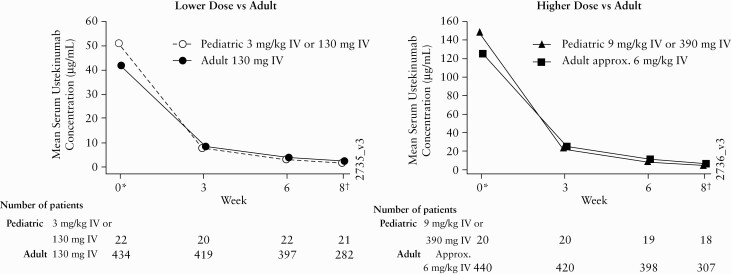
Lower and higher dose paediatric vs adult mean serum UST concentrations through Week 8. IV, intravenous; Ped, paediatric; UST, ustekinumab.

Through Week 6 in the overall paediatric population [combined doses], SUCs were generally comparable to those in the reference adult Phase 3 CD studies [NCT01369329, NCT01369342, and NCT01369355; Figure 3].

At Week 0 following infusion, Week 3, and Week 6, respectively, mean serum ustekinumab concentrations were 51.3 μg/mL, 7.7 μg/mL, and 3.0 μg/mL following the low induction dose in the overall paediatric population, compared with 41.9 μg/mL, 8.4 μg/mL, and 3.8 μg/mL following the 130-mg dose in adults. At the same time points, mean ustekinumab concentrations were 148.7 μg/mL, 23.7 μg/mL, and 9.1 μg/mL, respectively, following the high induction dose in the overall paediatric population, compared with 125.2 μg/mL, 25.2 μg/mL, and 11.1 μg/mL following the ~6-mg/kg dose in adults.

**Figure 4.**
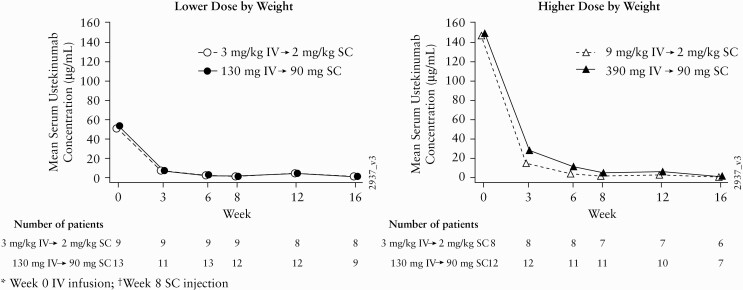
Summary of mean serum UST concentrations through Week 16 by body weight. BW, body weight; IV, intravenous; SC, subcutaneous; UST, ustekinumab; lower dose, 3 mg/kg IV for patients <40 kg BW or 130 mg IV for patients ≥40 kg BW; higher dose, 9 mg/kg IV for patients <40 kg BW or 390 mg IV for patients ≥40 kg BW.

In the higher dose group, a pattern toward lower mean SUC was observed in patients weighing <40 kg versus those weighing ≥40 kg **[**Figure 4**]**.

### 3.2. Clinical and endoscopic outcomes

**Figure 5.**
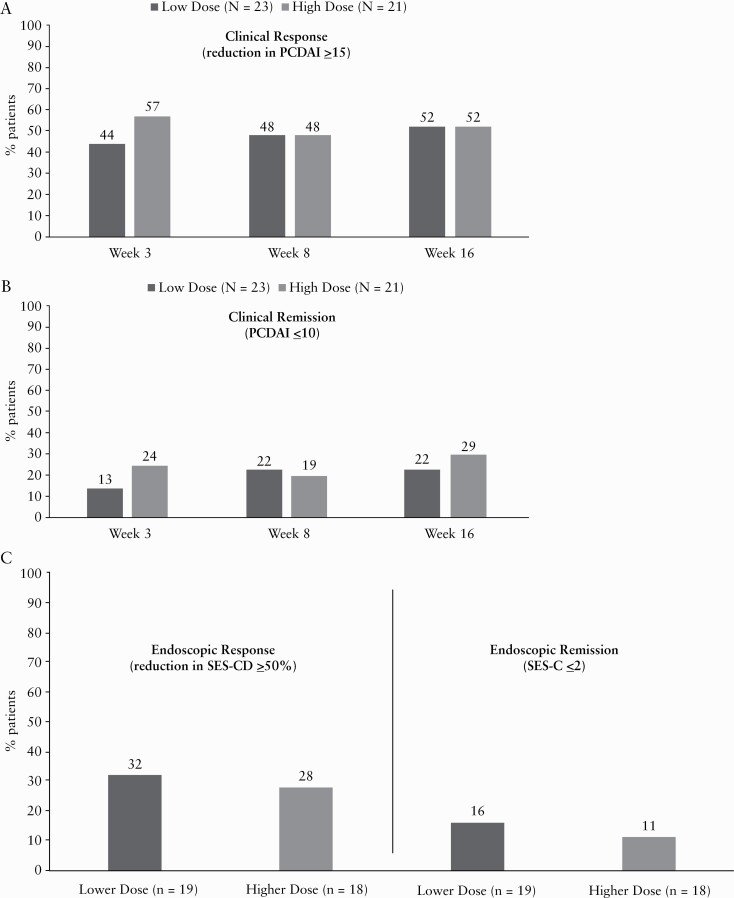

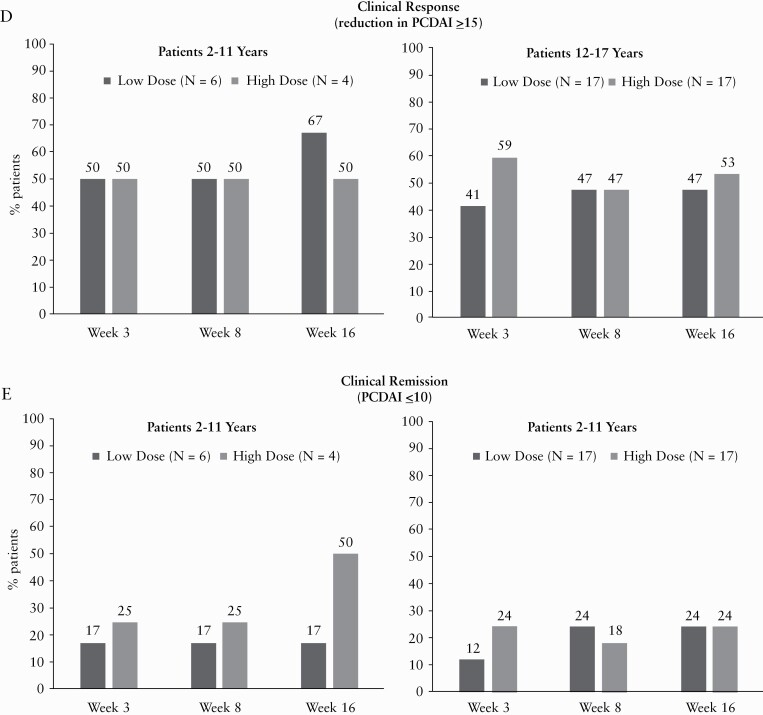

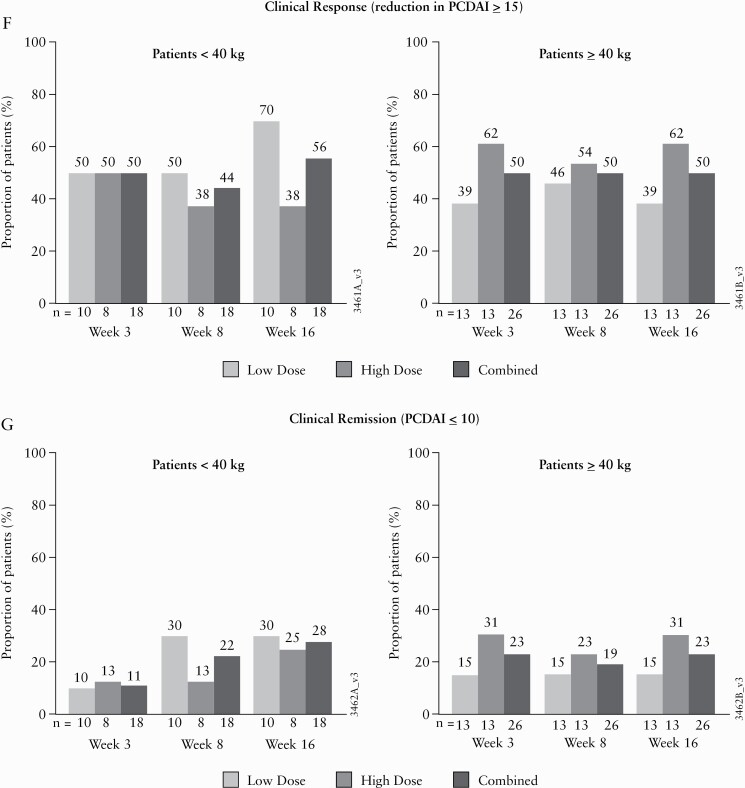
[a] Clinical response at Weeks 3, 8, and 16. [b] Clinical remission at Weeks 3, 8, and 16. [c] Endoscopic response and remission at Week 16. [d] Clinical response by age at Weeks 3, 8, and 16. [e] Clinical remission by age at Weeks 3, 8, and 16. [f] Clinical response by weight at Weeks 3, 8, and 16. [g] Clinical remission by weight at Weeks 3, 8, and 16. BW, body weight; IV, intravenous; PCDAI, Paediatric Crohn’s Disease Activity Index; SES-CD, Simple Endoscopic Score for Crohn’s Disease; low dose, 3 mg/kg IV for patients <40 kg BW or 130 mg IV for patients ≥40 kg BW; high dose, 9 mg/kg IV for patients <40 kg BW or 390 mg IV for patients ≥40 kg BW. ^a^Patients who had a prohibited Crohn’s disease-related surgery, or who discontinued study agent due to an adverse event of worsening Crohn’s disease or due to lack of efficacy or had prohibited concomitant medication changes, were considered not to be in endoscopic response or remission. In addition, patients with missing segments at the designated analysis time point had their baseline score for the missing segment[s] carried forward. Lower dose = 3 mg/kg IV for patients <40 kg BW or 130 mg IV for patients ≥40 kg BW; higher dose = 9 mg/kg IV for patients <40 kg BW or 390 mg IV for patients ≥ 40kg BW.

At Week 8, 48% of patients in the lower and higher dose groups were in clinical response; these rates were similar at Week 16 **[**Figure 5a]. The percentage of patients in clinical remission was greater in the higher than in the lower dose group at Weeks 3 and 16; at Week 8, remission rates were similar **[**Figure 5b]. Among the patients in clinical response at Week 8 [47.7%], the percentages of patients who maintained clinical response and achieved clinical remission at Week 16 were 81.0% and 38.1%, respectively, in the combined ustekinumab group. The proportions of patients achieving clinical response or clinical remission were generally consistent across baseline age and weight subgroups **[**Figures 5d–f].

Additional subgroup analyses were conducted to evaluate consistency of efficacy outcomes [clinical response and clinical remission at Week 8] by different baseline characteristics [demographics, baseline disease characteristics, and baseline concomitant medication]. Results were generally similar between subgroups and consistent with the overall study [data not shown].

Overall, patients had endoscopies at baseline and Week 16 [*n* = 37] where 32% and 28% of patients had an endoscopic response in the lower and higher dose groups and 16% [*n* = 3] and 11.1% [*n* = 2] had achieved endoscopic remission, respectively **[**Figure 5c].

### 3.3. Corticosteroid use

The mean steroid dose at baseline was 14.4 mg/day [fivepatients] for the low dose group and 10.6 mg/day [seven patients] for the high dose group. At Week 8, the mean steroid dose was 10.2 mg/day [five patients] for the low dose group and 10.4 mg/day [five patients] for the high dose group. At Week 16, the mean steroid dose was 45.0 mg/day [two patients] for the low dose group and 4.1 mg/day [four patients] for the high dose group. From baseline to Week 16, steroid use [including budesonide] declined in the low [30% to 13%] and high dose groups [33% to 19%; Table 2]. In the low dose group, patients [*n* = 5/23; 22%] were in steroid-free clinical remission at Weeks 8 and 16; in the high dose group, 2/21 [10%] and 5/23 [24%] were in steroid-free clinical remission at Weeks 8 and 16, respectively. For those who were on steroids at baseline and Weeks 8 and 16, median prednisone equivalent doses of steroids [excluding budesonide] increased for the low dose group [10.0, 10.0, and 45.0 mg/day] and decreased for the high dose group [8.0, 2.5, and 4.4 mg/day], respectively. Only 3

**Table 2.** Corticosteroid use.

	Patients receiving steroids, *n* [%]^a^		Steroid-free clinical remission, *n* [%]^b,c,d^	
	Low UST dose [*n* = 23]^e^	High UST dose [*n* = 21]^f^	Low UST dose^e^ [*n* = 23]	High UST dose [*n* = 21]^f^
Baseline	7 [30]	7 [33]	Na	Na
Week 8	7 [30]	5 [24]	5 [22]	2 [10]
Week 16	3 [13]	4 [19]	5 [22]	5 [24]

AE, adverse event; BW, body weight; CD, Crohn’s disease; IV, intravenous; Na, not assessed; PCDAI, Paediatric Crohn’s Disease Activity Index; UST, ustekinumab.

^a^Including budesonide.

^b^Patients who had a prohibited Crohn’s disease-related surgery or discontinued study agent due to an AE of worsening CD, or due to lack of efficacy, or had prohibited concomitant medication changes, were considered not to be in clinical remission and not receiving corticosteroids.

^c^Patients who had insufficient data to calculate the PCDAI score at that visit were considered not to be in clinical remission and not receiving corticosteroids.

^d^Patients who had a missing value in corticosteroid use at Week 8 [Week 16] had their last value carried forward.

^e^Low dose, 3 mg/kg IV for patients <40 kg BW or 130 mg IV for patients ≥40 kg BW.

^f^High dose , 9 mg/kg IV for patients <40 kg BW or 390 mg IV for patients ≥40 kg BW.

three patients in the low dose group and four patients in the high dose group were on steroids at Week 16. Patients who initiated or increased steroid dose up to Week 16 were considered treatment failures.

### 3.4. Inflammatory biomarkers

**Figure 6.**
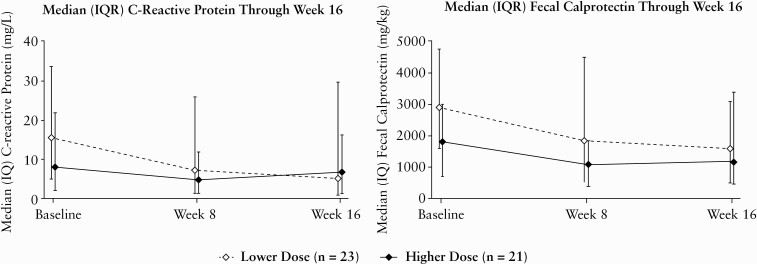
Inflammatory biomarkers through Week 16. BW = body weight; IQR, interquartile range; IV, intravenous; lower dose, 3 mg/kg IV for patients <40 kg BW or 130 mg IV for patients ≥40kg BW; higher dose, 9 mg/kg IV for patients <40 kg BW or 390 mg IV for patients ≥40kg BW.

At baseline, 32/44 or 72.7% of patients had abnormal levels of CRP. In the low dose group, 3/18 or 16.7% patients had normalised CRP levels at Week 8 and 3/18 or 16.7% had normalised CRP levels at Week 16. For the high dose group, 4/14 or 28.6% had normalised CRP levels at Week 8 and 3/14 patients or 21.4% had normalised levels at Week 16. The median [IQR] change from baseline at Week 8 in CRP concentration was -0.72 [-8.01; 0.12] mg/L for the lower dose group and -0.27 [-14.4; 0.30] mg/L for the higher dose group **[**Figure 6**]**. The median [IQR] change from baseline at Week 16 was 0 [-9.3; 0.04] mg/L for the lower dose group and -0.82 [-8.65; 0] mg/L for the higher dose group. The median [IQR] change from baseline at Week 8 in faecal calprotectin concentration was 0 [-2395.00; 418.00] mg/kg and -37.00 [-1347.00; 553.00] mg/kg for those in the lower and higher dose groups, respectively **[**Figure 6**]**. The median [IQR] change from baseline at Week 16 was 0 [-3438.00; 190.00] mg/kg and 0 [-1126.00; 654.00] mg/kg for patients in the lower and higher dose groups, respectively.

### 3.5. Clinical benefit and pharmacokinetics

**Figure 7.**
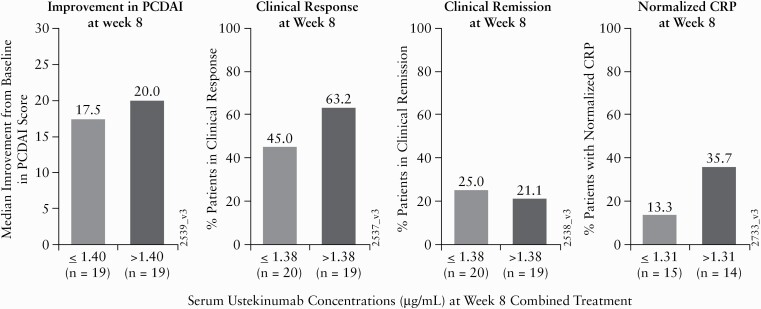
Clinical response, clinical remission, and median improvement from baseline in the PCDAI score at Week 8 by median serum ustekinumab concentrations [µg/mL] at Week 8 [PK Analysis Set]. CRP, C-reactive protein; PCDAI, Paediatric Crohn’s Disease Activity Index; PK, pharmacokinetics.

At Week 8, a greater proportion of patients were in clinical response in the higher ustekinumab concentration group compared with the lower ustekinumab concentration group **[**Figure 7**]**. Similar trends were observed for median improvement from baseline in the PCDAI score. However, no correlation was observed between SUC and clinical remission at Week 8.

### 3.6. Safety/tolerability

Through Week 16, 73% of patients reported one or more AEs **[**Table 3**]**. More AEs/serious AEs [SAEs] occurred in the lower dose group than the higher dose group at Weeks 8 and 16. SAEs occurred in 26% of patients [*n* = 6] in the lower dose group and 5% of patients [*n* = 1] in the higher dose group, with CD exacerbation being the most frequent SAE [9% and 5%, respectively].

**Table 3.** Safety/tolerability overview through Week 16.

*n* [%]	Lower UST dose^a^ [*n* = 23]	Higher UST dose^b^ [*n* = 21]	Combined [*n* = 44]
Deaths	0	0	0
Discontinued due to AE	1 [4]	1 [5]	2 [5]
Patients with ≥1 AE	19 [83]	13 [62]	32 [73]
AEs occurring in ≥10% of patients			
Worsening of Crohn’s disease	6 [26]	3 [14]	9 [21]
Anaemia	6 [26]	1 [5]	7 [16]
Headache	5 [22]	3 [14]	8 [18]
Upper RTI	3 [13]	2 [10]	5 [11]
Anal fistula	3 [13]	1 [5]	4 [9]
Pyrexia	3 [13]	1 [5]	4 [9]
Cough	3 [13]	0	3 [7]
Fatigue	1 [4]	3 [14]	4 [9]
Patients with ≥1 SAEs	6 [26]	1 [5]	7 [16]
Worsening of Crohn’s disease	2 [9]	1 [5]	3 [7]
Constipation	1 [4]	0	1 [2]
Intestinal obstruction	1 [4]	0	1 [2]
Small intestinal obstruction	1 [4]	0	1 [2]
Vision blurred	1 [4]	0	1 [2]
General physical health deterioration	1 [4]	0	1 [2]
Myalgia	1 [4]	0	1 [2]
Infections	9 [39]	8 [38]	17 [39]
Serious infections^c^	0	0	0
Neoplasms [malignant]	0	0	0
AEs during/<1 h of infusion	1 [4]	0	1 [2]
ISR at Week 8	0	0	0

AEs, adverse events; BW, body weight; ISR, injection site reactions; IV, intravenous; RTI, respiratory tract infections; SAEs, serious AEs; UST, ustekinumab.

^a^Lower dose, 3 mg/kg IV for patients <40 kg BW or 130 mg IV for patients ≥40 kg BW.

^b^Higher dose, 9 mg/kg IV for patients <40 kg BW or 390 mg IV for patients ≥40 kg BW.

^c^Through the final safety visit, one patient in the low dose group experienced an intestinal abscess which resolved without sequelae.

Other reported SAEs were intestinal obstruction, small intestinal obstruction, blurred vision, physical health deterioration, and myalgia. Of thetwo2 patients with reported intestinal bowel obstruction [these events were not considered treatment related], one patient resolved with surgery and one patient resolved without surgery. Infections [e.g., upper respiratory tract infection, anal abscess, *Clostridium difficile*, infected eczema, gastroenteritis, viral gastroenteritis, and nasopharyngitis] occurred in 39% of patients [lower dose: 39%, *n* = 9; higher dose: 38%, *n* = 8]; there were no serious infections through Week 16. However, through the final safety visit, one patient in the low dose group experienced intestinal abscess, which resolved with sequelae. No injection site reactions, anaphylaxis, or serum sickness-like events, opportunistic infections, malignancies, or deaths were reported, and no patients tested positive for antibodies to ustekinumab through Week 16. One AE, temporally related to infusion, was reported in the lower induction dose group. A patient had pyrexia with a temperature of 38.1°C within 1 h of an infusion. The administration was stopped, and the patient’s temperature returned to normal after a few minutes, so the administration was resumed.

## 4. Discussion

We conducted a study evaluating a range of ustekinumab doses in paediatric patients with moderate to severe CD. The primary objective of this study was met. Results show that serum concentrations of ustekinumab over time in the paediatric CD population were generally comparable to those in the reference adult CD population from the Phase 3 ustekinumab development programme **[**Figure 3**].** However, the subgroup analysis showed that SUCs were lower among children with body weight <40 kg compared with adolescents with body weight ≥40 kg and the reference Phase 3 adult population. This suggests that children with body weight <40 kg may require a different dose regimen from that employed in this study to attain similar exposures of ustekinumab compared with the typical adult population receiving the 390 mg induction dose.

With respect to immunogenicity, using a drug-tolerant assay, no patients had detectable antibodies to ustekinumab through Week 16, a finding consistent with the low incidence of immunogenicity in the reference adult CD population. Overall, results suggest that the PK and immunogenicity profiles are generally similar between paediatric and adult patients with CD and suggest that the higher dose induction regimen evaluated in this study, intended to provide ustekinumab exposures in paediatric patients commensurate with exposures observed in adult patients with CD, is appropriate in children ≥40 kg.

At Week 8, ustekinumab induction treatment resulted in meaningful improvements in the efficacy endpoints evaluated, including PCDAI-based clinical response and clinical remission. Although modest, overall improvements in objective measures of inflammation [reductions from baseline in CRP and faecal calprotectin] were generally reflective of improvements in efficacy outcomes.

Overall, improvements in efficacy outcomes were considered generally similar between the two treatment groups and were observed as early as Week 3. Importantly, whereas definitive conclusions on the two dose regimens cannot be made based on efficacy results alone, the overall benefit profile observed was consistent with benefits observed in the adult CD studies.

Baseline disease characteristics were representative of a paediatric population of patients with moderately to severely active CD, with the majority having failed one or more anti-TNF agents. Overall, demographic and baseline characteristics including median duration of disease and median PCDAI score, a major efficacy variable, were generally well balanced across the two treatment groups. Although the proportions of patients with abnormal levels of inflammatory markers at baseline were similar across treatment groups, overall baseline values for these and SES-CD measures were noted to be somewhat greater for patients in the lower than in the higher induction dose group. Notably, whereas some heterogeneity, largely attributable to the limited sample size, was observed in baseline values, overall inflammatory burden suggested by CRP and faecal calprotectin was generally consistent with that observed in the ustekinumab adult CD programme.

Ustekinumab induction and short-term maintenance treatment through Week 16 were generally well tolerated in this paediatric CD population. Although SAEs and CD events were higher in the lower dose group, there was no evidence of an increased risk of infections in either group. But again, the rate of CD-related AEs in the lower dose group is notable. There were no injection site reactions, and one AE temporally related to infusion was reported in the lower induction dose group. No anaphylactic or serum sickness-like reactions were reported. Although interpretation is limited by small sample sizes, no patterns were observed suggesting a different safety profile in the younger or smaller [<40 kg] participants compared with the older participants [aged 12 to 17 years] or those ≥40 kg. Overall, safety and tolerability results from this study suggest that the safety profile of ustekinumab in the paediatric CD population was generally consistent with the established safety profile of ustekinumab in the adult CD population.

In conclusion, improvements in clinical and endoscopic disease activity were observed across the efficacy outcome measures evaluated. Reductions were seen in objective biomarkers of inflammation in this treatment-refractory group of children with CD. The pharmacokinetics and safety profiles were consistent with those reported for adults with CD treated with ustekinumab. Overall, our data favour a dosing regimen in paediatric patients ≥40 kg like that approved for patients aged 18 years or older. Additional PK analyses are needed in the patients under 40 kg.

## Supplementary Material

jjab089_suppl_Supplemental_Figure_1

## Data Availability

The data sharing policy of Janssen Pharmaceutical Companies of Johnson & Johnson is available at [https://www.janssen.com/clinical-trials/transparency]. As noted on this site, requests for access to the study data can be submitted through Yale Open Data Access [YODA] Project site at [http://yoda.yale.edu].
