# Supplementary figures and images for: Ustekinumab in Paediatric Patients with Moderately to Severely Active Crohn’s Disease: Pharmacokinetics, Safety, and Efficacy Results from UniStar, a Phase 1 Study
Source: J Crohns Colitis. 2021 May 26;15(11):1931–42. doi: 10.1093/ecco-jcc/jjab089 (PMC8575045; doi:10.1093/ecco-jcc/jjab089)

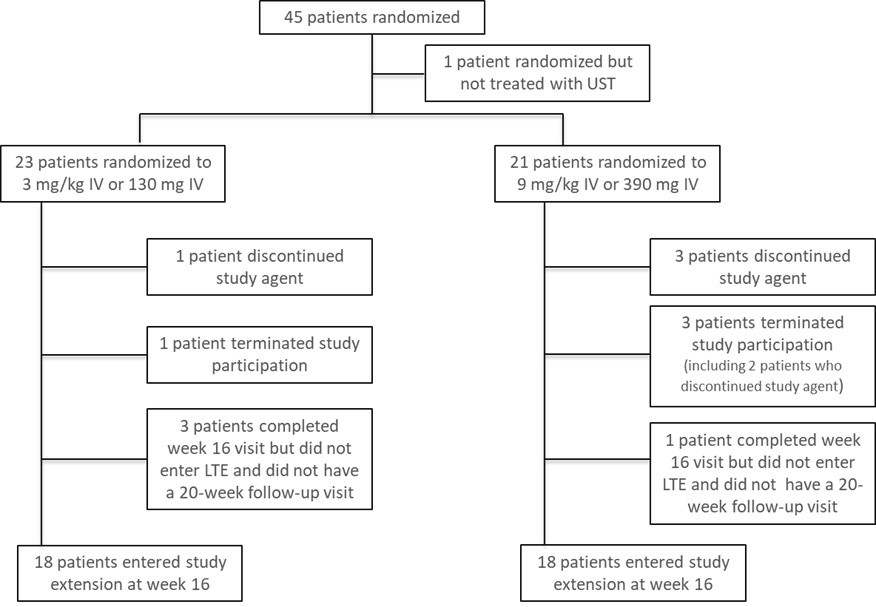

Supplement: jjab089_suppl_Supplemental_Figure_1 [file jjab089_suppl_supplemental_figure_1.jpeg]
